# The Molecular Regulatory Mechanism in Multipotency and Differentiation of Wharton’s Jelly Stem Cells

**DOI:** 10.3390/ijms241612909

**Published:** 2023-08-18

**Authors:** Li Ma, Xuguang He, Qiang Wu

**Affiliations:** The State Key Laboratory of Quality Research in Chinese Medicine, Macau University of Science and Technology, Macau 999078, China

**Keywords:** WJ-MSCs, transcriptional regulation, epigenetic modifications, signaling pathways, differentiation

## Abstract

Wharton’s jelly-derived mesenchymal stem cells (WJ-MSCs) are isolated from Wharton’s jelly tissue of umbilical cords. They possess the ability to differentiate into lineage cells of three germ layers. WJ-MSCs have robust proliferative ability and strong immune modulation capacity. They can be easily collected and there are no ethical problems associated with their use. Therefore, WJ-MSCs have great tissue engineering value and clinical application prospects. The identity and functions of WJ-MSCs are regulated by multiple interrelated regulatory mechanisms, including transcriptional regulation and epigenetic modifications. In this article, we summarize the latest research progress on the genetic/epigenetic regulation mechanisms and essential signaling pathways that play crucial roles in pluripotency and differentiation of WJ-MSCs.

## 1. Introduction

WJ-MSCs were originally isolated as fibroblast-like cells from human umbilical cords by McElreavey [1]. Subsequent studies found that the surface of these cells express CD105, CD73, and CD90 surface markers of mesenchymal stem cells [2]. They can differentiate into bone, cartilage, and fat cells [3]. These features meet the standard of pluripotent mesenchymal stem cells established by the International Society for Cell Therapy in 2006 [4]. Therefore, WJ-MSCs have attracted massive attention as an important source of multipotent stem cells. Interestingly, pluripotency markers such as OCT4, NANOG, SOX2, and MYC are expressed in WJ-MSCs [5]. Thus, WJ-MSCs have some characteristics of both embryonic stem cells (ESCs) and adult stem cells and may be a bridge between these two types of cells [6]. WJ-MSCs are convenient to isolate and there are no ethical disputes associated with their use. Importantly, WJ-MSCs are not tumorigenic and have strong cell proliferation, differentiation, and immune regulation capabilities [7]. Therefore, WJ-MSCs have become more and more important in applications of mesenchymal stem cells. Due to these advantages, studies on WJ-MSCs and related applications have gradually increased. Hence, it is very important to study the molecular regulatory mechanisms of their biological characteristics.

Epigenetic regulation is the reversible and heritable change of gene expression levels by altering the topological structure of chromatin and regulating transcription initiation, elongation, DNA replication, and DNA repair without modifying the gene sequence [8]. Epigenetic regulation is mainly achieved through DNA methylation, histone modifications, and non-coding RNAs [9]. Epigenetic modification plays a fundamental regulatory role in the process of determining the fate of stem cells, exerting influence at multiple levels pre-, mid-, and post-transcription. Thus, epigenetic modification is an endogenous regulatory mechanism in stem cell fate decisions [10].

Gene expression directs protein synthesis and cellular functions. One of the mechanisms that regulates the complex relationship between genes and their products is transcriptional regulation inside the cell. Transcription factors (TFs) can bind to a specific gene sequence through their DNA binding domain and assist RNA polymerase II and TFII to form an effective transcription initiation complex, thereby initiating gene expression [11]. TFs play a central role in determining cell differentiation fate throughout embryonic development and maintaining the cellular properties of adult tissues [12]; the expression of even a single TF can determine the direction of cell differentiation [13].

Epigenetic regulation and transcriptional regulation can explain the unique biological characteristics of WJ-MSCs at the molecular level. Therefore, this article concisely summarizes the research progress on the epigenetic and transcriptional regulation mechanisms that maintain the properties of WJ-MSCs. We also briefly discuss the related signaling pathways that play an important role in WJ-MSCS.

## 2. Epigenetic Regulation

### 2.1. DNA Methylation

DNA methylation is catalyzed by DNA methyltransferases (DNMTs), using S-Adenosyl methionine (SAM) as a methyl donor, and transferring a methyl group to cytosine phosphate guanine dinucleotide co-transferring to CpG islands. The process results in the formation of 5-methylcytosine (5mC) through valent combination [14,15]. DNA demethylation is the process of removing a methyl group from cytosine through active or passive demethylation processes. TET (Ten-eleven translocation) is mainly involved in the active demethylation process since it can demethylate DNA by oxidizing 5mC to an unmethylated cytosine intermediate [16,17]. These two types of enzymes, DNMTs and TETs, play an important role in the process of DNA methylation/demethylation, thus regulating the maintenance, proliferation, differentiation, and apoptosis of stem cells by activating or repressing gene expression.

DNMT3A is highly expressed in differentiated cells and is involved in DNA de novo methylation, and hence it plays an important role in normal development [18]. EID3 can repress transcription and inhibit cell differentiation [19]. Studies have shown that EID3 can directly affect DNMT3A in WJ-MSCs, thereby directing MSCs to differentiate [20]. In mice, Tet2 can regulate the cell cycle of MSCs [21]. Tet1 and Tet2 can ensure the self-renewal and differentiation of MSCs by maintaining the demethylation status of the *p2rx7* promoter [22]. TET1 and TET2 play the same role in human MSCs, and they affect the function of MSC differentiation by regulating lineage-determining genes [23]. Interestingly, TET1 and TET2 have different expression patterns in MSCs from different sources [24]. However, whether TET plays the same role in WJ-MSCs has not been reported and further exploration is needed.

The methylation status of the promoter regions of pluripotency genes such as *OCT4* and *NANOG* can limit the differentiation potential of MSCs, while the methylation status of lineage-specific genes can also affect the differentiation potential of MSCs [25]. Hypermethylation in adipose, bone marrow, and muscle-derived MSCs hinders differentiation, whereas hypomethylation status has no predictive value for differentiation potential [26,27]. However, hypomethylation in WJ-MSCs has an impact on differentiation potential. Studies have shown that hypomethylation in the promoter regions of cardiac lineage-specific factors *GATA4*, *TBX5*, *SERCA*, and *NKX2.5* in WJ-MSCs correlates with differentiation potential. Due to the hypomethylation status of cardiac lineage-specific factors in WJ-MSCs, they are ideal cell sources for differentiation into cardiomyocytes [28]. In WJ-MSCs, adipose differentiation is also associated with hypomethylation status, and the decrease of CpG methylation in the *MEST* promoter may promote the binding of SOX6, thereby enhancing the expression of *MEST* and stimulating the differentiation of WJ-MSCs into adipocytes [29]. It is noteworthy that this difference between WJ-MSCs and MSCs derived from other sources may also be regulated by DNA methylation [30].

### 2.2. Histone Modifications

Histones are the basic structural proteins in eukaryotic chromatin and are mainly divided into H2A, H2B, H3, and H4. Histones can be modified in a variety of ways, such as histone methylation, acetylation, phosphorylation, ubiquitination, and ADP-ribosylation [31]. These diverse histone modifications can dynamically switch chromatin states from transcriptionally active to transcriptionally silent, and vice versa.

**Figure 1:**
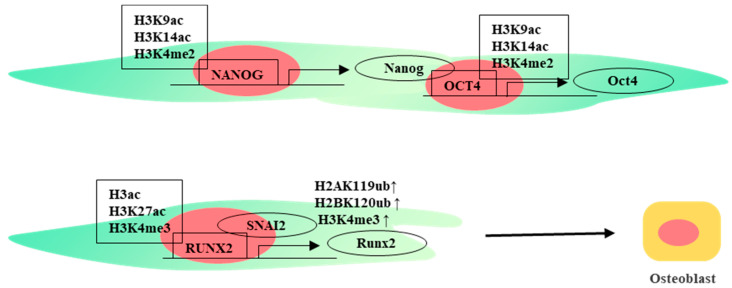
Histone modifications in differentiation of WJ-MSCs.

It has been found that the changes of histone H3K9ac and histone H3K4me2 in the *OCT4* and *NANOG* promoters were consistent with the corresponding gene expression levels in WJ-MSCs. By affecting histone H3K9 or H3K14 acetylation and histone H3K4 dimethylation, the mRNA expression of pluripotency-related genes and proliferation genes can be increased and the spontaneous differentiation of MSCs can be inhibited [32]. RUNX2 is an important factor in regulating osteogenic differentiation [33], and the P1 promoter region of *RUNX2* regulates the transcription of *RUNX2*/*p57* which are the main regulators of osteogenic differentiation [34]. In undifferentiated WJ-MSCs, the P1 promoter region of *RUNX2* lacks the histone marks H3ac, H3K27ac, and H3K4me3, which are associated with transcriptionally active genes. When the histone marks H3ac, H3K27ac, and H3K4me3 were enriched in the P1 promoter region, RUNX2/P57 gene expression was activated and WJ-MSCs differentiated toward osteoblasts [35]. H3K4 methylation, H3K27 acetylation, and DNA demethylases also have a strong correlation with upregulation of gene expression, and they can jointly regulate gene expression [36] (Figure 1).

Histone modification is inseparable from the action of related enzymes. Histone deacetylases (HDACs) mediate the deacetylation of histone, compacting chromatin, impeding the ability of transcription factors to bind to DNA, and thus inhibiting gene transcription. There are currently 18 HDACs found in humans, which are mainly divided into 4 types [37]. In embryonic stem cells, inhibition of HDACs reduces the self-renewal capacity of the cells and increases the expression of differentiation markers. Likewise, HDACs are also critical for maintaining the self-renewal and differentiation of MSCs. It has been reported that inhibition of HDACs, histone H3, and H4 acetylation can block the G2/M phase of cells, inhibiting self-renewal, increasing the expression of osteogenic genes, and thus directing MSCs to differentiate into osteoblasts [38,39]. However, this is contrary to other reports [32]. The discrepancy may be due to the use of different cell types or different HDAC inhibition methods, which may produce different effects. Interestingly, inhibition of HDAC in WJ-MSCs can reduce cell proliferation [37] and promote cardiomyocyte formation by upregulating NKX2.5 [40], and inhibition of HDAC IIa can induce WJ-MSCs to differentiate into pancreatic endocrine-like cells [41].

### 2.3. Non-Coding RNA

Non-coding RNAs play an irreplaceable role in basic physiological processes including development, immunity, and reproduction. There are more than 100 known RNA modifications, many of which can participate in the regulation of eukaryotic gene expression.

miRNAs are a class of small, endogenous, non-coding single-stranded RNAs. miRNAs target the 3′-UTR region of mRNAs. As a result, the mRNAs are degraded or translation is inhibited, thereby affecting the protein expression level [42]. Thus, miRNAs play a crucial role in post-transcriptional regulation [43]. miRNAs are involved in many biological processes such as cell proliferation, differentiation, apoptosis, and migration, and can also participate in the regulation of stem cell pluripotency [44,45]. In WJ-MSCs, miR-196b-5p can block the G0/G1 phase, inhibit cell proliferation, and promote collagen content in the extracellular matrix. Meanwhile, this miRNA can regulate the downstream key gene *SERPINB2* of osteogenic differentiation and stimulate osteogenic ability, thus promoting new bone formation [46,47]. miRNAs also play an important role in the differentiation of WJ-MSCs into neurons [48]. In mice, overexpression of miR-3099 can up-regulate genes related to neuronal differentiation: *NeuN*, *Tuj1*, *NeuroD1*, *Sox4*, *Gat1*, *vGluT1*, and *vGluT2*, and down-regulate genes related to astrocyte generation process including *Gfap*, *S100β*, and *Slc1a3*, thus playing a key role in controlling the expression of key neuronal plasticity markers and in the regulation of learning and memory. In humans, MDS21, which is highly expressed in WJ-MSCs, targets similar genes as miR-3099 [49]. This may be one of the reasons why WJ-MSCs are more advantageous in neuron differentiation. miR-20b and miR-106a can directly or indirectly regulate the expression of neuronal genes to affect neural differentiation by inhibiting the expression of *NGN2*, *MAP2*, and *TUBB3* [50]. In WJ-MSCs, depletion of endogenous miR-34a expression results in enhanced motility of WJ-MSCs, which may contribute to neuronal precursor motility [51]. SOX11 and VIM can maintain the undifferentiated state of WJ-MSCs. During the differentiation of WJ-MSCs into hepatocytes, miR-122-5p can bind to the 3′-UTR regions of *SOX11* and *VIM* and inhibit the expression of *SOX11* and *VIM*, thus promoting the differentiation of WJ-MSCs into hepatocytes by down-regulating mesenchymal marker genes [52]. In addition, miR-146a-5p is essential for the motility and proliferation of MSCs. In WJ-MSCs, knockdown of miR-146a-5p can inhibit cell proliferation and enhance cell migration [53].

CircRNAs are novel non-coding RNA molecules that do not have a 5′ cap and 3′ poly(A) tail and are formed by covalent bonds creating a ring structure. CircRNAs are species-, tissue-, and time-specific and can regulate cell differentiation, tissue homeostasis, and disease development. CircRNA contains miRNA binding sites and can function as a miRNA sponge, thus indirectly regulating the expression of downstream target genes of miRNA. In the process of stem cell differentiation, many circRNAs are differentially expressed, and the stem cell differentiation of different lineages can be regulated through the circRNA–miRNA–mRNA interaction network. Hence, CircRNA can be used as a biomarker and target for stem cell therapy [54]. In WJ-MCSs, circRNA also plays an important role. For instance, CircRNA_05432, circRNA_08441, and circRNA_01536 are related to cell proliferation and differentiation and play a role in the differentiation of WJ-MSCs into cardiomyocyte-like cells [55]. CDR1as also participates in the proliferation and differentiation of WJ-MCSs, regulates the cell cycle and apoptosis of WJ-MSCs, and affects the expression of stem transcription factors, which plays an important role in pluripotency regulation. Interestingly, circRNA can also influence the therapeutic effect of stem cells. For example, circ6401 binds to miR-29b-1-5p in WJ-MSCs and regulates the repair of the endometrium by affecting the VEGF signaling pathway [56].

LncRNAs are non-coding RNAs with a length of more than 200 nucleotides and have no ability to encode proteins. They can participate in the methylation process of CpG islands [34] and the recruitment of related essential molecules to methylated or ubiquitinated proteins, hence playing roles in epigenetic regulation [57]. LncRNA are important factors in the regulation of MSC function [58]. lncRNA *CIR* can bind to EZH2 and inhibit the expression of ATOH8 and the chondrogenic differentiation of WJ-MSCs through EZH2-mediated H3K27me3 [59]. *ITGA6-AS1* is the antisense non-coding RNA of ITGA6. ITGA6 can maintain the self-renewal of stem cells. The post-transcriptional regulation of *ITGA6* in WJ-MSCs is controlled by *ITGA6-AS1*. Therefore, the regulation of *ITGA6-AS1* may be associated with the cell identity of WJ-MSCs [60].

**Figure 2:**
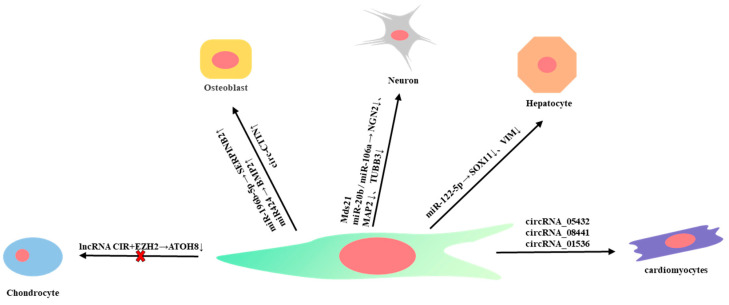
Noncoding RNAs affect cell differentiation of WJ-MSCs.

Interestingly, non-coding RNA can interact with DNA methylation and histone modification to form a precise network of gene regulation. Oct4 overexpression can downregulate miR-148a through DNA methylation. miR-148a can directly regulate DNMT1 by targeting the 3′-UTR of its transcript. Thus, reduced expression of miR-148a can induce high levels of DNMT1 [44]. OCT4 and NANOG can also directly bind to the *DNMT1* promoter to increase the expression of DNMT1. Increased expression levels of DNMT1 can then lead to repressed expression of genes associated with development and lineage differentiation [61] (Figure 2).

## 3. Transcriptional Regulation

Transcriptional regulation changes the level of gene expression by altering the rate of transcription. Transcription factors (TFs) play a vital role in the mechanism of regulating transcription. TFs regulate chromatin structure and gene transcription by recognizing specific DNA sequences and play an important role in gene expression, cell differentiation and development, autophagy, metabolism, and apoptosis [62]. Compared with other types of stem cells, WJ-MSCs have a unique transcriptional profile. Genes related to the immune system, chemotaxis, and cell death are highly expressed in WJ-MSCs [63]. The formation of this unique transcriptional expression profile is highly associated with the intracellular TF function in WJ-MSCs.

OCT4, SOX2, and NANOG are key transcription factors regulating ESC pluripotency and reprogramming [64,65]. It is interesting that undifferentiated ESC markers can also be detected in WJ-MSCs. These genes include *OCT4*, *SOX2*, *NANOG*, *LIN28*, *SSEA1*, *SSEA3*, *SSEA4*, *KLF4*, *MYC*, *CRIPTO*, *REX1*, *TDGF1*, and *ZFP42* [63,66]. Although the expression levels of OCT4, NANOG, SOX2, and LIN28 are low in WJ-MSCs [63], their expression remains detectable despite different isolation methods and preservation techniques [67]. These suggest that WJ-MSCs have similar primitive, undifferentiated properties with ESCs.

In ESCs, the pluripotency and self-renewal of ESCs are maintained through the “Oct4-centered” and “Myc-centered” transcriptional regulatory networks [68]. In MSCs, OCT4 can target genes similar to those in ESCs, promote the expression of MSCs-specific genes, and regulate the pluripotency of MSCs through similar regulatory mechanisms [69]. OCT4 can bind to the SOX2 promoter to initiate SOX2 transcription. The expression level of SOX2 in WJ-MSCs is much higher than that of OCT4 [70], and SOX2 is considered to be an important factor regulating the pluripotency and cell cycle of WJ-MSCs [71]. The transcription of SOX2 is regulated by a variety of transcription factors. In addition to OCT4, the transcription of SOX2 is also regulated by E2F1. After E2F1 binding to the *SOX2* promoter, the expression of SOX2 increases and cell stemness is enhanced [72]. SOX2 can regulate cell growth by inhibiting CCND1 and CDK4 [73]. In WJ-MSCs, E2F1, as a transcription factor, can regulate the expression of SOX2. It also binds to open chromatin structures containing histone H3K27ac and H3K4me3 and activates ELOVL2 expression, leading to impaired mitochondrial oxygen utilization function and affecting cell metabolism [74].

NR2F2 (nuclear receptor family F group 2) is mainly expressed in mesenchymal cells. NR2F2 is an important regulator of differentiation and is related to tissue homeostasis. NR2F2 has been considered to be a key regulator of mesenchymal stem cell fate. The regulation of NR2F2 on stem cell fate cannot be separated from the role of miRNA. MiR-194 directly targets the 3′-UTR of NR2F2 mRNA to negatively regulate NR2F2 expression, thus regulating bone and fat formation [75]. NR2F2 also affects the formation of cartilage, and MSCs overexpressing NR2F2 show significantly enhanced cartilage formation [76]. In WJ-MSCs, NR2F2 can also regulate the expression of CCND1 and CDK4, affecting cell proliferation and inflammatory factor secretion of WJ-MSCs, and has potential roles in the treatment of diseases such as immunity and cancer [77].

p53 is a key transcription factor involved in the regulation of cell differentiation, cell stemness, and development [78]. Loss of p53 leads to accelerated proliferation and differentiation of MSCs, which are more inclined to differentiate into adipocytes and osteoblasts [79,80]. Some studies have shown that p53 inactivation plays an active role in stemness maintenance, as knocking out p53 in MSCs increases the expression of stemness markers such as OCT4, SOX2, and NANOG [81]. It is noteworthy that p53 can directly or indirectly inhibit the functions of important transcription factors in stem cells, and thus the ultimate biological outcome of p53 is dependent on the specific functions of those transcription factors in specific stem cell types [82]. p53 can also bind to *OSX* and prevent OSX and transcriptional cofactors from binding to Sp1/GC-rich sequences in OSX, thereby inhibiting OSX transcription. OSX is an important transcription factor regulating osteogenic differentiation, and its transcriptional repression directly affects osteogenic differentiation [83].

SNAI2 (formerly known as Slug) is a zinc finger transcription factor that is usually considered to play a role in the process of epithelial–mesenchymal transition. Studies have found that transcription factors related to epithelial–mesenchymal transition also regulate the functions of embryonic and adult stem cells, which are also the main regulatory factors for stem cell function and cell differentiation [84]. The interaction between Snail/Slug and YAP/TAZ controls the self-renewal and differentiation characteristics of mesenchymal stem cells [85]. During the differentiation of WJ-MSCs into osteoblasts, overexpression of SNAI2 can promote the deposition of cellular mineralized matrix [86].

HNF4A is a transcriptional regulator that controls the expression of liver-related genes during the transformation of endodermal cells to hepatic progenitor cells and is also associated with stem cell differentiation. In WJ-MSCs, overexpression of HNF4A can improve the hepatic differentiation capacity of WJ-MSCs [87]. Hence, overexpression of HNF4A in WJ-MSCs may be a better choice to treat acute liver failure [88].

Taken together, although some transcription factors have been shown to be important in regulating the biological characteristics of WJ-MSCs, systematic screening for essential transcription factors that are crucial for WJ-MSCs has been lacking until recently. Therefore, research which aims to decode the transcription network that governs cell WJ-MSCs’ fate will be prominent in the near future.

## 4. Signaling Pathways

**Figure 3:**
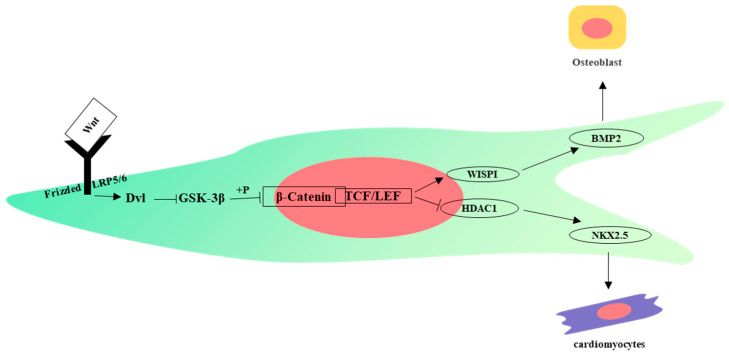
Wnt signaling pathway influences differentiation of WJ-MSCs.

The Wnt pathway regulates key developmental processes and tissue homeostasis by coordinating cell proliferation, differentiation, cell polarity, cell motility, and stem cell renewal. The Wnt signaling pathway can be divided into the Wnt/β-Catenin pathway, Wnt/PCP pathway, and Wnt/Ca^2+^ pathway. The first type is a canonical Wnt signaling pathway, and the latter two are non-canonical Wnt signaling pathways [89]. Interestingly, the Wnt signaling pathway can regulate the self-renewal and differentiation of MSCs [90]. Differences in the expression of Wnt signaling-related molecules affect the osteogenic differentiation of MSCs. Compared with BM-MSCs, the Wnt signaling inhibitor DKK1 is upregulated in WJ-MSCs, thus the Wnt/β-Catenin pathway is inhibited and the expression of its downstream target gene *WISP1* is lower. The decreased expression of WISP1 may be the reason why the ability of WJ-MSCs to differentiate into osteoblasts is not as good as that of BM-MSCs [91]. The Wnt signaling pathway also promotes cardiomyocytes but inhibits cardiomyocyte differentiation at later stages of development. WNT signaling represses HDAC1 expression through the β-catenin/LEF1 pathway, resulting in decreased HDAC1 expression and increased NKX2.5 expression and differentiation toward cardiomyocytes, but it has the opposite effect at late stages [92] (Figure 3).

**Figure 4:**
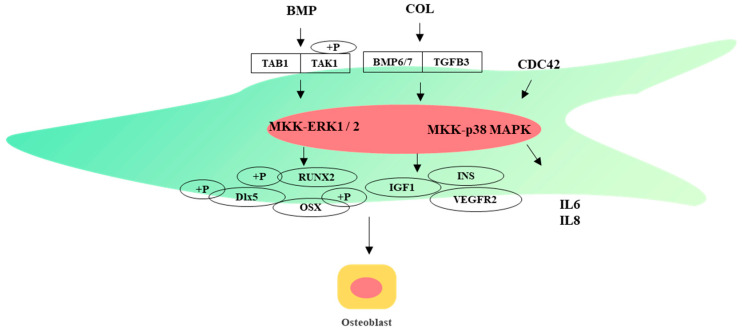
MAPK pathway directs WJ-MSCs into osteoblasts.

MAPKs are a group of evolutionarily conserved serine–threonine kinases, which can be divided into four subfamilies: ERK, p38, JNK, and ERK5, representing four different cascade pathways, respectively. The MAPK pathway can be activated by extracellular stimuli such as cytokines, cellular stress, hormones, and neurotransmitters. After the upstream activator protein binds to a specific receptor, it activates its downstream transcription factors through the gradual phosphorylation of MAP3K–MAP2K–MAPK, and then regulates diverse cellular functions including gene expression, cell proliferation, migration, differentiation, and stress response in MSCs [93,94,95]. In WJ-MSCs, CDC42 can affect the proliferation of WJ-MSCs and the secretion of inflammatory factors IL-6 and IL-8 by affecting the phosphorylation of ERK1/2 and p38 MAPK [96]. IL-1β can induce the expression of MMP-3 and enhance the migration of WJ-MSCs through ERK1/2, JNK, and p38 MAPK signaling pathways [97]. IGFBP2 promotes adipogenic differentiation of WJ-MSCs by increasing the phosphorylation of JNK/AKT and activating the JNK/AKT signaling pathway [98]. COL induces osteogenic differentiation by activating the transcription of BMP6/7 and TGFB3, and then activating the downstream target genes *INS*, *IGF1*, *RUNX2* and *VEGFR2* through the p38/MAPK pathway [99]. After BMP receptors are activated, phosphorylated TAK1 recruits TAB1 to initiate MKK-p38 MAPK or MKK-ERK1/2 signaling (Figure 4).

In WJ-MSCs, the SHH pathway, NF-kB pathway, and STAT pathway also play important roles. The SHH pathway can stimulate and promote the production of angiogenic factors such as activin A, angiopoietin, angiopoietin 1, granulocyte-macrophage colony-stimulating factor, matrix metalloprotein peptidase-9, and urokinase-type plasminogen activator. These factors can be secreted to enhance the pro-angiogenic ability of WJ-MSCs in vitro and in vivo [100]. Activation of the NF-kB pathway can protect WJ-MSCs from serum deprivation-induced apoptosis [101]. After the NF-kB pathway is inhibited, its downstream iNOS and cytokine signaling are inhibited, and the antioxidant enzyme system is enhanced. Hence, the anti-inflammatory effect of WJ-MSCs can be better exerted [102]. The STAT pathway can mediate the immunosuppression of B7-H1 induced by IFN-γ [103].

## 5. Other Important Molecules in Differentiation of WJ-MSCs

WJ-MSCs have strong differentiation ability and can be induced into different types of cells through different induction methods. Some key factors play an important role in this process.

JARID1B (Jumonji AT-rich interactive domain 1B) is a histone demethylase that can specifically remove the H3K4me3 mark and inhibit transcription of its related genes, playing an important role in cell fate determination, cancer progression, and stem cell self-renewal role [104]. In WJ-MSCs, the activity of JARID1B can regulate the expression of RUNX2 and affect the differentiation ability of osteoblasts.

KGF (keratinocyte growth factor) is a member of the fibroblast growth factor family. KGF is produced by mesenchymal cells and exerts its biological effects by binding to their receptors with high affinity. KGF plays an important role in regulating embryonic development, cell proliferation, and cell differentiation. Studies have shown that KGF can affect the proliferation of pancreatic ductal cells through the MEK-ERK1/2 pathway and induce the differentiation of pancreatic ductal cells into β cells through the PI3K/AKT pathway [105]. KGF is also a key growth factor for the differentiation of WJ-MSCs into sweat gland-like cells [106], although the specific mechanism has not yet been uncovered.

NGF (Nerve growth factor) is a neurotrophic factor that induces neurite outgrowth and promotes the survival and maintenance of neurons. NGF, its precursor molecule pro-NGF, and its different receptor systems, such as TrkA, p75NTR, and sortilin, play key roles in the development and physiological functions of the nervous and immune systems. The combination of NGF and TrkA can activate Ras and downstream MAPK pathways [107]. Interestingly, overexpression of NGF in WJ-MSCs will affect the expression levels of SOX1, SOX2, and NES, showing that overexpression of NGF can lead to the differentiation of MSCs into neural cells [108]. BDNF (Brain derived neurotrophic factor) is also an important neurotrophic factor that can guide cells into the pathway of neuronal differentiation [109]. BDNF activates the ERK pathway by binding to specific Trk receptors and participates in cell differentiation [110].

## 6. Combined Factors Mediate Osteogenic Differentiation of WJ-MSCs

To date, a great deal of efforts have been put into directing WJ-MSCs to differentiate into osteoblast for clinical applications. Intriguingly, the differentiation of WJ-MSCs into osteoblasts involves different genetic/epigenetic regulatory mechanisms that work together to promote WJ-MSCs to differentiate into osteoblasts. For instance, RUNX2, a downstream target gene of the p38/MAPK signaling pathway [99], is also an important regulator of osteogenic differentiation. When JARID1B is enriched in the P1 promoter region of *RUNX2*, the levels of activation marks, including histone H3ac and H3K4me3, are reduced, whereas increased levels of the suppressive mark histone H3K27me3 lead to the inhibition of *RUNX2* transcription [34]. On the contrary, when JARID1B is inactivated, histone marks H3ac, H3K27ac, and H3K4me3 are enriched in the P1 promoter region, and SNAI2 also binds to the RUNX2 promoter to activate RUNX2 expression [111].

In addition, coupled with the activation of the MAPK signaling pathway, the transcriptional activity of RUNX2 can be further increased after phosphorylation [112]. Furthermore, RUNX2 can mediate the BMP-2-induced expression of OSX [113]. BMP2 is regulated by the downstream target genes *WISP1* [114] and *miR424* [115] of the Wnt signaling pathway, which enhances its induction of OSX. OSX is another important regulator of osteogenic differentiation. When the promoter region of *OSX* is bound by p53, its transcription is inhibited, thus affecting osteogenic differentiation [83]. Conversely, when p53 is inactivated, H2BK120ub1 and H3K4me3 are enriched in the *OSX* promoter region, and *OSX* transcription can be increased [116]. At the same time, the activation of the MAPK signaling pathway can also increase the transcriptional activity of *OSX* [112] and promote the transcription of downstream osteogenic genes *OPN*, *OCN*, and *COL1A1*. During osteogenic differentiation, the increased activity of H2AK119ub, H2BK120ub, and H3K4me3 [116], and the participation of *CDR1as* [117] and *circ-CTTN* [118] can jointly promote the differentiation of osteoblasts. Hence, many factors can induce WJ-MSCs to differentiate into osteoblasts.

## 7. Conclusions and Prospects

WJ-MSCs have broad application prospects due to their unique biological characteristics. Their molecular features are regulated by complicated epigenetic mechanisms, specific transcription factors, and signaling pathways. However, our current understanding of WJ-MSCs is still very limited since the majority of research has been focused only on their clinical applications. As basic research on WJ-MSCs is still relatively limited, further exploration of regulatory mechanisms of WJ-MSCs by utilizing single-cell analysis and omics methods should be conducted. The new knowledge gained will surely fully harness the great potential of WJ-MSCs in regenerative medicine.

## Data Availability

Not applicable.
